# Responses of the soft coral *Xenia elongata* following acute exposure to a chemical dispersant

**DOI:** 10.1186/s40064-015-0844-7

**Published:** 2015-02-13

**Authors:** Michael S Studivan, Walter I Hatch, Carys L Mitchelmore

**Affiliations:** Department of Biology, St. Mary’s College of Maryland, 18952 E. Fisher Rd, 20686 St. Mary’s City, MD USA; Chesapeake Biological Laboratory, University of Maryland Center for Environmental Science, PO Box 38, 20688 Solomons, MD USA; Present Address: Harbor Branch Oceanographic Institute, Florida Atlantic University, 5600 N US Highway 1, 34946 Fort Pierce, FL USA

**Keywords:** Xenia elongata, Coral bleaching, Oil dispersant, Corexit 9500, Percent symbiont loss

## Abstract

Limited toxicology data are available regarding oil dispersant exposure to coral species. Corexit® EC9500A (Corexit) is a commonly applied dispersant most well known for its use after the Deepwater Horizon spill in April, 2010. There is limited evidence that Corexit can cause a bleaching response in corals. The aims of the study were: (1) to determine the extent of bleaching after acute 24 h and 72 h exposures of sublethal concentrations (0-50 ppm) of Corexit to the pulsing soft coral *Xenia elongata* and (2) to investigate a percent symbiont loss calculation using zooxanthellae density. The percent symbiont loss calculation was compared to a traditional metric of normalizing zooxanthellae density to soluble protein content. Percent symbiont loss was an effective measure of coral stress in acute Corexit exposures, while protein normalized zooxanthellae density was more variable. The bleaching data suggest a positive relationship between dispersant concentration and percent symbiont loss, culminating in excessive tissue necrosis and coral mortality within 72 h in high concentration exposures (p < 0.001). Percent beaching ranged from 25% in 5 ppm exposures to 100% in 50 ppm exposures. Corexit also caused a significant decrease in pulse activity (p < 0.0001) and relative oxygen saturation (p < 0.001), possibly indicating a reduction in photosynthetic efficiency. This study and other similar research indicate that dispersant exposure is highly damaging to marine organisms, including ecologically important coral species.

## Background

The ecological effects of oil dispersant exposure on coastal ecosystems are still unknown despite dispersant application in oil spill cleanup for the past 50 years. Dispersants break up surface oil slicks into small droplets that are more miscible with water to distribute the oil throughout the entire water column (Fiocco and Lewis 1999). Dispersal reduces oil concentrations at the surface, but increases distribution in the water column, possibly affecting diverse populations of pelagic and benthic organisms (Schmidt 2010). Dispersants have been found to increase the availability of microscopic oil droplets to hydrocarbon-degrading bacteria (Leahy and Colwell 1990; Hamdan and Fulmer 2011; Brakstad 2008), yet compounds such as Corexit® EC9500A (herein Corexit) has recently been shown to reduce the viability of several species of microbes, including hydrocarbon-degraders (Brakstad 2008; Hamdan and Fulmer 2011). Corexit has been prominently used as an oil dispersant for the past 30 years, including its most extensive application to date during the Deepwater Horizon spill cleanup. Quantitative data on the consequences of dispersant exposure on many taxa, most notably fish, invertebrates, and coral species, is lacking. The Deepwater Horizon spill cleanup was the first mass use of dispersants at depth and knowledge about exposure on deep water communities, including coral reefs, is equally limited (Kujawinski et al. 2011). Deep injection of dispersants ultimately leads to more dispersal time in the water column, where contact with biological habitats may be prolonged.

The effects of oil exposure on corals have been relatively well-documented following previous spills (Fishelson 1973; Loya and Rinkevich 1980, 1979; Peters et al. 1981; Cook and Knap 1983; Cohen et al. 1977; Guzman et al. 1994). Documentation of dispersant toxicity, conversely, is comparatively poor, even with commonly used compounds. Very few field studies and measurements exist for oil dispersants alone, as the Deepwater Horizon spill cleanup was one of the first mass application of dispersants. Detection of Corexit in marine environments is often limited to quantification of its chemical constituents, but concentrations are generally highest within the first few meters. Bocard *et al.* (1984) observed 13 ppm dispersant concentrations in open sea trials, and concentrations up to 19 ppm were measured in coastal waters seven months after the start of the Deepwater Horizon accident (Hayworth and Prabakhar Clement 2012). Higher concentrations may be possible on shallower coral reef environments with less water flow than the open ocean. Toxicological data indicates that Corexit is not a significant threat to model invertebrate and fish species within environmentally relevant concentrations (Environmental Protection Agency 1995). However, a hydra species was far more sensitive to Corexit, with LC50 values an order of magnitude lower than the EPA model species (27 ppm vs. 2.3 ppm, respectively) (Mitchell and Holdway 2000). Other cnidarians appear to be similarly sensitive to dispersant exposure, therefore, one research priority is to compare stress responses among the EPA test species to other marine species including corals. Preliminary experiments with the soft coral *Xenia elongata* exposed to concentrations as low as 20 ppm of Corexit resulted in tissue disintegration, complete zooxanthellae expulsion, and eventual colony death. Shafir et al. (2007) observed higher levels of mortality in *Stylophora pistillata* and *Pocillopora damicornis* fragments exposed to dispersed oil compared to oil alone. Also, dispersants become more toxic to coral larvae than oil by damaging tissue and reducing settlement rates (Epstein et al. 2000; Goodbody-Gringley et al. 2013). Dispersed oil led to reduced photosynthesis rates in zooxanthellae within *Diploria strigosa* colonies, implying that the coral-algal symbiosis is affected (Cook and Knap 1983).

Under normal circumstances, zooxanthellae reside symbiotically within coral endoderm tissue, providing 95% of the coral’s metabolic need via photosynthesis and enhanced calcification in exchange for protection, nutrients, and carbon dioxide (Muscatine 1990). When a coral becomes stressed, a breakdown of the coral-algal symbiosis may occur, where the coral host expels resident zooxanthellae in a process known as bleaching. Bleaching has been relatively well described with several mechanisms of zooxanthellae release known, and a multitude of environmental conditions and stressors may disrupt the symbiosis (Brown 1997; Glynn 1996; Lesser 2004; Gates et al. 1992). A variety of stressors including metals, oil, and pesticides may cause expulsion of zooxanthellae in coral tissue (Jones 1997; Brown 2000). As coral bleaching events have become more prominent worldwide, bleaching has been more commonly used as a measure of coral health (Brown 1997; Fitt et al. 2001; Glynn 1996; Glynn et al. 2001; Meehan and Ostrander 1997). Previous measurements of bleaching have used noninvasive techniques that approximate the extent of bleaching with presence or absence of bleached tissue, color reference cards, or visual percentage of pigment loss (Siebeck et al. 2006; Shafir et al. 2007; Glynn et al. 2001). Studies designed to quantify the extent of bleaching may provide additional important information about: (1) the effect of stressors on coral health and recovery, (2) the mechanism of bleaching, and (3) the ecological effects of oil dispersants in spill cleanup. This study reflects on a relatively novel technique used to assess the severity of bleaching in a simple dispersant dose experiment.

*Xenia elongata* was chosen as a suitable test organism for this study as the species was readily available, fast growing, and easily cloned. This species is a soft coral of the Xeniidae family (Cnidaria, Anthozoa, Alcyonacea) commonly found on shallow Indo-Pacific reefs (Fabricius and De’ath 2008). *Xenia elongata* is sensitive to changes in water quality and may serve as a bioindicator for coral species in other locations. Since this species directly absorbs organic compounds from the water, it is likely to show rapid responses to direct dispersant contact (Sprung and Delbeek 1997). Additionally, related species have been used as model organisms in previous oil and dispersant research, including preliminary research by the authors (Cohen et al. 1977; Epstein et al. 2000). The unique polyp pulsing observed in Xeniid corals (rhythmic movement of polyp’s tentacles) is predicted to be a mechanism to reduce oxidative stress on the colony and increase photosynthesis, through facilitation of oxygen exchange with the water (Morgan 2010; Kremien et al. 2013). If Corexit exposure affects photosynthetic rates of the zooxanthellae, a stress response may be demonstrated through decreased pulse activity (Cohen et al. 1977).

Although oceanographic conditions prevented the Deepwater Horizon spill from reaching shallow water coral reefs, the results of this experiment contribute to the growing understanding of dispersant effects on coral species for future dispersant applications. We tested several concentrations of Corexit® EC9500A (0 ppm, 5 ppm, 20 ppm, and 50 ppm) for 24 h and 72 h periods to model acute exposures. *Xenia elongata* was predicted to demonstrate stress responses when exposed to increasing dispersant concentrations, measured by proportional expulsion of the symbiotic zooxanthellae and changes in the pulse activity of the colony. Bleaching severity was measured through a quantification of percent symbiont loss (Jones 1997; Perez et al. 2001), calculated using the following formula:

$$ \frac{\mathbf{expelled}\ \mathbf{zooxanthellae}}{\left(\mathbf{expelled}\ \mathbf{zooxanthellae}+\mathbf{host}\ \mathbf{zooxanthellae}\right)} \times 100\% $$

By quantifying percent symbiont loss, normalizing zooxanthellae counts to surface area, protein content, or biomass may not be necessary. Hard coral surface area can be calculated relatively easily with aluminum foil, measurement of tissue area, or computer modeling (Edmunds and Gates 2002; Naumann et al. 2009). These methods are simple and have demonstrated success in hard coral studies, but they fail to account for varying tissue depth and become less precise with complex surface geometries. Some soft corals are dependent on a hydraulic skeleton reinforced by spicules and therefore are more variable in size and shape than hard corals (Sprung and Delbeek 1997; Hellström and Benzie 2011). Most traditional normalization methods cannot be used with soft corals as a result, except for protein content. However, previous research indicates that coral protein concentration may be variable and is therefore not as accurate for zooxanthellae loss normalization as other metrics (Kendall Jr et al. 1983; Edmunds and Gates 2002). This experiment compared a traditional measure of zooxanthellae density normalized to protein content to the percent symbiont loss calculation. One would expect to see an inverse relationship between the two metrics, where percent symbiont loss would increase with Corexit exposure and zooxanthellae density per mg/ml protein would decrease**.** Lastly, measurement of percent symbiont loss allows for comparison between cnidarian bleaching responses regardless of species or skeleton.

## Materials and methods

All applicable international, national, and/or institutional guidelines for the care and use of animals were followed. *Xenia elongata* colonies were cloned from genotypically identical parent colonies maintained at St. Mary’s College of Maryland (St. Mary’s City, MD). Cloned *X. elongata* colonies were propagated on plastic trays in flow-through seawater from May 2010 to May 2011 when not exposed to dispersant at a mean temperature of 25°C, salinity of 35 ppt, and reef spectrum fluorescent lighting for 14 h a day. The mean light intensity was 11,100lux. Four concentrations of Corexit modeling zero (0 ppm), low (5 ppm), moderate (20 ppm), and heavy exposure (50 ppm) were prepared based on previous dispersant exposure studies and a dispersed oil spill trajectory model (Goodbody-Gringley et al. 2013; Wetzel et al. 2001; Mearns et al. 2003). The dilution factor of each solution was verified using a spectrophotometer measurement at 220 nm. Six colony replicates were randomly selected for each concentration and treatment time, and were randomly distributed in individual 1000 mL beakers within two identical plastic bins. Replicates were between 7-10 cm in fully extended height with 20-30 polyps. In order to maintain a stable temperature, the bins were half submerged in the system. The need for artificial aeration was tested in preliminary trials by measuring dissolved oxygen over a 72 h period in aerated and nonaerated beakers, but was deemed not necessary to maintain a minimum oxygen content in the dispersant-exposed water.

Coral colonies were exposed to Corexit solutions in two independent time treatments of 24 h and 72 h to determine if dispersant exposure resulted in compounding stress responses over time. Water was not changed during the experimental treatment, so as to not lose any expelled zooxanthellae prior to sample collection and quantification. The original experimental objectives involved exposure times of 24 h and 96 h, but all preliminary exposed colonies died after 72 h. Qualitative observations were made based on colony appearance 1 h after initial dispersant exposure and throughout the experiment. Pulsing characteristics were assessed at 24 h for both time treatments and at 72 h for the 72 h treatment only. Pulse rate was quantified by counting three polyps per colony for 20 seconds (pulses per minute). We judged pulse intensity of all colonies on a scale of 0-4 (0 = no movement or complete polyp balling, 1 = slight movement or polyp squirming, 2 = moderate movement or slight pulsing, 3 = movement and some polyp extension, 4 = full movement and polyp extension). Relative oxygen saturation was measured every four to eight hours starting at 24 h post initial dispersant exposure as a rough estimation of photosynthetic stress. All corals were consumptively sampled at the end of their respective exposure times for quantification of percent symbiont loss.

At the end of each exposure period, the culture water was homogenized with an immersion blender in order to dislodge zooxanthellae adhering to the beaker. Agitated culture water was subsampled and centrifuged to isolate expelled zooxanthellae. Each colony was placed into separate tubes and then immediately homogenized using a tissue grinder continuously for a two-minute period. Tissue samples were centrifuged to pellet the zooxanthellae and coral tissue was removed with a vacuum aspirator. Two zooxanthellae subreplicate counts of each sample were completed using a hemacytometer. For samples with necrosis, zooxanthellae in the necrotic tissue fragments could not be counted, as cells were extremely dense in several focal planes. However, the presence of necrotic tissue on the hemocytometer was qualitatively noted. Bleached and remaining zooxanthellae densities were calculated to determine the total number per experimental colony. Zooxanthellae density data was also normalized to measurements of soluble protein content in the coral host using the standard BioRad protein assay (Bradford 1976). Statistical analyses were performed in SAS 9.3 (Cary, NC). Percent symbiont loss data were arcsine-square root transformed and analyzed using a two-way ANOVA and Tukey post hoc tests to compare Corexit doses between the exposures times. Zooxanthellae density normalized to protein content data were analyzed using the same statistical tests. Pulse rate and relative oxygen saturation data were analyzed for a significant effect of dispersant concentration and exposure time with repeated measures ANOVAs. Pulse intensity data were analyzed for the 24 h and 72 h treatments separately using two independent samples Kruskal-Wallis tests. Alpha was set at 0.05.

## Results

**Figure 1:**
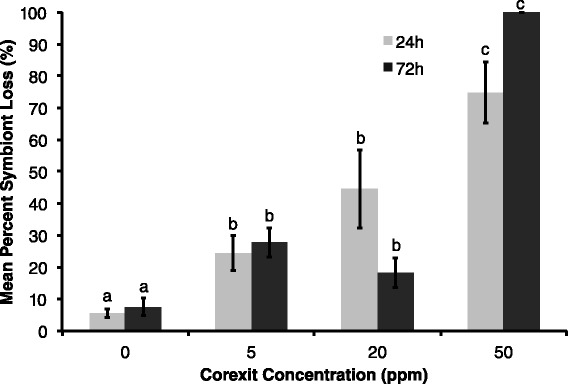
**Percent symbiont loss with dispersant exposure; Mean percent symbiont loss of** ***Xenia elongata*** **exposed to increasing concentrations of Corexit for 24 h and 72 h periods (F = 31.08, p < 0.0001), where error bars represent one standard error of the mean and different letters denote significant difference between means.**

**Figure 2:**
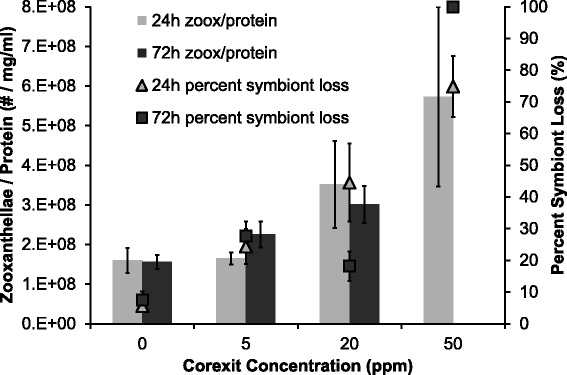
**Percent symbiont loss vs. zooxanthellae density normalized to soluble protein; Percent symbiont loss (points) compared to zooxanthellae density per mg/ml protein (bars), where error bars represent one standard error of the mean.**

There was a significant increase of percent symbiont loss with increasing Corexit concentration in both exposure times (F = 31.08, p < 0.0001). Figure 1 presents the untransformed percent symbiont loss data, while statistical analyses were performed on the arcsine square root transformed data. Corexit concentration alone had a significant effect on the severity of bleaching (F = 63.46, p < 0.0001). Exposure time did not have an overall significant effect on percent symbiont loss, however there was a significant interaction between dispersant concentration and exposure time (F = 8.85, p < 0.0001), likely due to coral death in the 72 h 50 ppm treatment. The mean percent symbiont loss was unexpectedly low in the 72 h 20 ppm treatment (18.2%) compared to the 24 h 20 ppm treatment (44.6%). All *X. elongata* colonies in the 72 h 50 ppm exposure died approximately 48 h after initial exposure, leaving no collectible tissues, and were therefore quantified as completely bleached (100%). Zooxanthellae density normalized to protein content was not significantly (but marginally) affected by dispersant exposures (F = 2.06, p = 0.0864), where only Corexit concentration had an effect (F = 3.25, p = 0.0346) and exposure time did not. When compared to the percent symbiont loss data, there was a similar increasing trend in the zooxanthellae per mg/ml protein data (Figure 2). Lines of best fit were calculated for each exposure time (24 h and 72 h), respectively. Using 0% symbiont loss as the baseline and 100% as the maximum, the EC_50_ was 28.01 ppm for the 24 h exposure and 25.47 ppm for the 72 h exposure.

$$ \boldsymbol{y}=1.2211\boldsymbol{x}+15.79 $$

$$ \boldsymbol{y}=1.2211\boldsymbol{x}+15.79 $$

**Figure 3:**
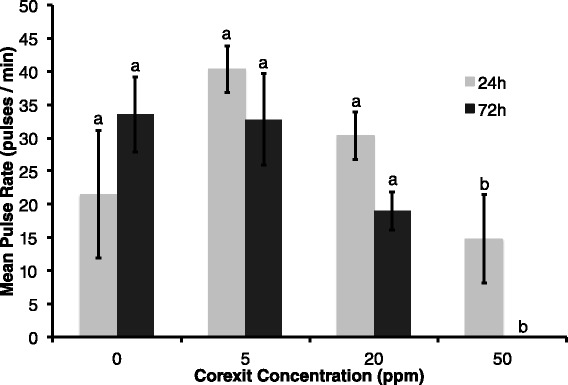
**Pulse rate with dispersant exposure; Mean pulse rate for 24 h (measured at 24 h) and 72 h (measured at 72 h) exposures with increasing dispersant concentration, where error bars represent one standard error of the mean and different letters denote significant difference of means (F = 23.97, p < 0.0001).**

**Figure 4:**
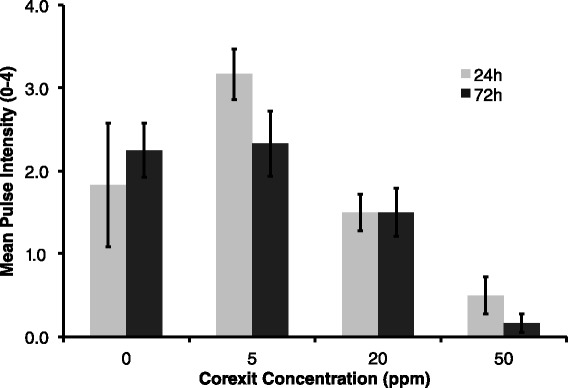
**Pulse intensity with Dispersant exposure; Mean pulse intensity for 24 h and 72 h exposures with increasing dispersant concentration, where error bars represent one standard error of the mean (p = 0.009 and p < 0.001, respectively).**

**Figure 5:**
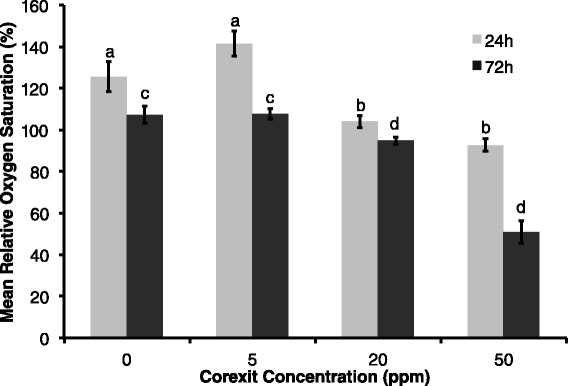
**Relative oxygen saturation with dispersant exposure; Mean relative oxygen saturation for 24 h (measured at 24 h) and 72 h (measured at 24 h, 30 h, 45 h, 50 h, 56 h, 68 h, and 72 h) exposures with increasing dispersant concentration, where error bars represent one standard error of the mean.**

**Figure 6:**
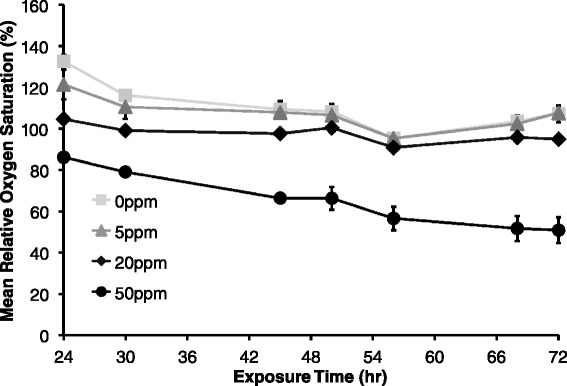
**Relative oxygen saturation between 24 and 72 h post dispersant exposure; Mean relative oxygen saturation for 72 h (measured at 24 h, 30 h, 45 h, 50 h, 56 h, 68 h, and 72 h) exposures with increasing dispersant concentration, where error bars represent one standard error of the mean and different letters denote significant difference of means (F = 67.48, p < 0.0001).**

Corexit concentration had a significant negative effect on pulse rate at all exposure times (F = 23.97, p < 0.0001). Additionally, pulse rate decreased over time in each Corexit concentration treatment group (F = 6.98, p = 0.0156), meaning pulse rate was lower in the 72 h exposure at 72 h compared to the 24 h and 72 h exposures measured at 24 h (Figure 3). Pulse intensity at the end of both time trials (24 h at 24 h and 72 h at 72 h) decreased significantly with higher exposure doses of Corexit (p = 0.009 and p < 0.001, respectively) (Figure 4). Relative oxygen saturation was negatively affected by Corexit concentration at all exposure times (F = 67.48, p < 0.0001) (Figure 5). Oxygen saturation also decreased over time (F = 47.22, p < 0.0001), most notably within 24 hours and between 56 and 72 hours, but there was a significant interaction between dose and time (F = 42.91, p = 0.0026) (Figure 6).

**Figure 7:**
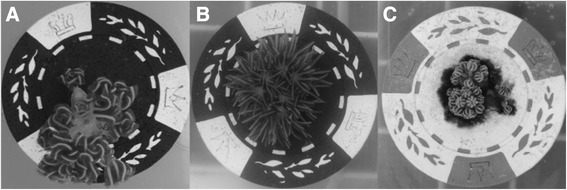
**Typical polyp behavior after dispersant exposure; A) Control colony with normal polyp appearance B) Abnormal polyp extension 1 h after initial dispersant treatment C) Expelled zooxanthellae around the base of a colony and balled polyps after 24 h.**

**Figure 8:**
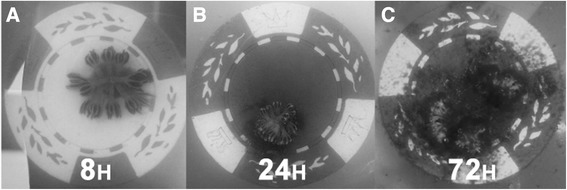
**Typical bleaching timeline after dispersant exposure; Typical timeline of colony appearance in 50 ppm dispersant treatment A) Loss of hydrostatic pressure after 8 h B) Bleaching and tissue necrosis after 24 h C) Decomposition of tissue and colony death after 72 h.**

Control colonies generally resumed normal pulse activity within 1 h of placement in experimental beakers (Figure 7A). Those exposed to dispersant immediately began showing signs of increased stress through decreased pulse activity. In the low dispersant application of 5 ppm, colonies had lower pulse rates, including some with balled polyps (Figure 7C). Approximately 1 h following initial exposure, colonies in higher concentrations (20 ppm and 50 ppm) ceased all movement with polyps fully extended (Figure 7B). After 8 h, signs of bleaching were apparent in 20 ppm and 50 ppm treatments. Most expelled zooxanthellae appeared as dark brown clumps adjacent to the coral base (Figure 7C). Colonies exposed to 50 ppm of dispersant lost hydrostatic pressure in the coenenchyme after 8 h (Figure 8A). At the end of the 24 h exposure, most 5 ppm colonies exhibited relatively healthy appearances and pulse activity. Colonies exposed to 20 ppm of Corexit had visibly bleached and showed depressed pulsing rates. Some 50 ppm colonies had begun to decompose after severe tissue necrosis occurred (Figure 8B). Entire polyps occasionally sloughed off 50 ppm colonies during removal from the beakers, indicating dead coral tissue. Similar but more pronounced effects were seen in the 72 h dispersant exposure. There was full necrosis, death, and subsequent decomposition of all colony tissue in the 50 ppm treatment (Figure 8C). After 72 h, there was no collectible tissue available in any of the 50 ppm colonies, as the corals had died.

## Discussion

Compared to previous toxicological data, this species appears to be similarly sensitive to dispersants as the EPA model species and previously studied organisms (Environmental Protection Agency 1995; Goodbody-Gringley et al. 2013). Concentrations as low as 5 ppm caused a percent symbiont loss of approximately 25% for both exposure times. Higher concentrations resulted in irreversible tissue damage and mortality (100% symbiont loss). The severity of bleaching that results in irreversible stress and eventual death of *X. elongata* colonies appears to be at or lower than 75%. Our findings suggest that the threshold bleaching value and lethal dispersant dose may be even lower if tissue necrosis occurs (Glynn et al. 1985; Glynn and D’croz 1990). When combined with EC_50_ values, it appears that concentrations of Corexit at or above 25 ppm result in severe coral bleaching. This does not mean, however, that dispersant concentrations below 25 ppm do not harm corals. Other effects of dispersant exposure were shown to disrupt coral tissues and alter normal behavior.

There appeared to be two methods of zooxanthellae expulsion seen following dispersant exposure: individual zooxanthellae were ejected from the coral host via exocytosis (evidenced by the lack of attached coral tissue), or necrosis, where dead tissue containing unexpelled zooxanthellae sloughed off the colony (Gates et al. 1992). Relatively large pieces of necrotic coral tissue containing hundreds to thousands of zooxanthellae were common in the higher exposure treatments. Necrosis was likely to have caused an underestimation of the percent symbiont loss calculation, as zooxanthellae contained within the necrotic tissue could not be quantified. Since sublethal tissue necrosis was most often observed in the 72 h 20 ppm exposure where mean percent symbiont loss was lower than expected, we predict necrosis to be a likely culprit. Sublethal necrosis may have been present and gone unnoticed in higher dispersant exposure treatments, as bleaching was probably more rapid and severe, or the colonies died before sampling. Similar tissue degeneration was observed in heavily bleached corals after an El Niño warming event in Panama (Glynn et al. 1985). Necrosis most likely has a substantial negative effect on typical bleaching responses. Whereas a colony may survive a mild bleaching event and eventually reuptake zooxanthellae (Buddemeier and Fautin 1993; Thornhill et al. 2006), tissue necrosis prevents recovery and generally leads to colony mortality (Glynn et al. 1985; Rodolfo-Metalpa et al. 2005; Dunn et al. 2002).

While Corexit concentration elicited a strong bleaching response in *X. elongata*, exposure time did not appear to increase bleaching. Responses to sublethal dispersant concentrations may be latent, therefore requiring a longer amount of time to observe compounded effects (Mitchell and Holdway 2000). When exposed to an acute (24 h) exposure to dispersant, *S. pistillata* nubbins experienced continued and increasing mortality up to 43 days post initial exposure (Shafir et al. 2003). Epstein et al (2000) observed delayed mortality of coral larvae 96 h after initial exposure. The significant interaction between dispersant concentration and exposure time in the percent symbiont loss data presented here may provide additional support for this claim, particularly for the higher Corexit concentrations.

While there was a similar increasing trend with increasing Corexit exposure in both percent symbiont loss and zooxanthellae density normalized to protein, only percent symbiont loss showed a significant change. The symbiont loss calculation presented in this study is more sensitive than the traditional method of normalizing to protein concentration. Corexit exposure may have affected the protein concentration as well within the corals, but this effect is relatively unstudied (Kendall Jr et al. 1983). The increasing trend observed with the protein-normalized zooxanthellae data may be explained by this claim. As bleaching severity increases, the value from the zooxanthellae per mg/ml protein metric should decrease as the numerator value decreases. However, if Corexit somehow negatively affected the protein concentration, the denominator value would decrease as well, possibly resulting in an increasing relationship with Corexit exposure.

Overall, since colony death may be unavoidable in dispersant dosing experiments, percent symbiont loss is a more practical approach than measuring protein. For the colonies in the 72 h 50 ppm treatment that died and decomposed, percent symbiont loss was recorded as 100%, while the lack of collectible coral tissue prevented protein from being quantified and therefore zooxanthellae density could not be normalized. Both metrics may have their advantages, but percent symbiont loss appears to be a viable option for soft coral species such as *Xenia elongata*, allowing comparison to other coral species and coral model species (Perez et al. 2001). In this particular study, percent symbiont loss was the only metric tested that produced usable data for all exposure treatments regardless of whether the colony survived.

Pulse rate decreased as expected, as pulsing in this species may be related to photosynthetic and respiratory efficiency through a facilitation of rapid oxygen diffusion (Morgan 2010). The mean pulse rate decreased in experimental colonies after exposure to Corexit, indicating that the photosynthetic rate of the zooxanthellae likely decreased or coral respiration increased (Cook and Knap 1983). We also observed an overall decrease in relative oxygen saturation as dispersant concentration increased and over time, which corroborates with a similar effect on coral photosynthesis or respiration, or possibly an increase in microbial respiration. Considering that microbes live symbiotically in coral tissue as well as in reef waters, the reported decrease in oxygen saturation could be a compounded result of both processes occurring simultaneously. Reduction in pulse rate may also have been a physiological response to mitigate the severity of bleaching. Corals that balled up their polyps (Figure 7C) appeared to drastically reduce the surface area of coral tissue exposed to Corexit*.* The zooxanthellae may be more protected and therefore less likely to be expelled.

This study reported the bleaching response of *Xenia elongata* when dosed with Corexit for acute exposures of 24 h and 72 h. The shallow water soft coral exhibited quantifiable and significant bleaching responses at the lowest dispersant concentration tested. Our data supports the use of the percent symbiont loss calculation in acute exposure studies as a metric for assessing coral stress, which appeared to be more sensitive than a traditional normalization of zooxanthellae density to protein concentration. The calculation may also be used to compare stress responses between different species in order to determine relative tolerance levels. There is still more information needed to fully understand the effects of oil dispersants on corals. This study did not incorporate oil or dispersed oil into the stress exposures, and toxicity data indicates that dispersed oil is far more toxic than oil or dispersant alone (Environmental Protection Agency 1995; Shafir et al. 2007; Epstein et al. 2000; Goodbody-Gringley et al. 2013). Studies that expose corals to low dispersant concentrations (0-5 ppm) would also be informative as these levels pertain to relevant concentrations distant from the spill site (Atwood and Ferguson 1982). Perhaps one of the most interesting results of this experiment is that dispersant exposure did not necessarily cause a straightforward bleaching response via zooxanthellae expulsion; instead some exposures caused tissue necrosis. While coral bleaching can be a reversible process, necrosis generally resulted in coral death and would obviously prevent recovery or reuptake of the zooxanthellae. The presence of necrosis in the coral stress response indicates that the bleaching process following dispersant exposure is a complex mechanism and that a multitude of responses should be examined in dispersant exposure studies. In order to better understand this process, differential gene expression profiling would be especially useful to identify the regulation of stress response genes following exposure (Edge et al. 2008; Kenkel et al. 2013). Given that oil spill cleanups involving dispersants often involve short periods of highly concentrated chemicals in the water prior to dispersal, care should be taken to avoid dispersant use near coral reefs.
